# Identification of Primary Antimicrobial Resistance Drivers in Agricultural Nontyphoidal Salmonella enterica Serovars by Using Machine Learning

**DOI:** 10.1128/mSystems.00211-19

**Published:** 2019-08-06

**Authors:** Finlay Maguire, Muhammad Attiq Rehman, Catherine Carrillo, Moussa S. Diarra, Robert G. Beiko

**Affiliations:** a Faculty of Computer Science, Dalhousie University, Halifax, Nova Scotia, Canada; b Guelph Research and Development Center, Agriculture and Agri-Food Canada (AAFC), Guelph, Ontario, Canada; c Canadian Food Inspection Agency (CFIA), Ottawa, Ontario, Canada; University of California San Diego

**Keywords:** AMR prediction, *Salmonella*, antimicrobial resistance, food chain, genomics, machine learning

## Abstract

Nontyphoidal *Salmonella* (NTS) is a leading global cause of bacterial foodborne morbidity and mortality. Our ability to treat severe NTS infections has been impaired by increasing antimicrobial resistance (AMR). To understand and mitigate the global health crisis AMR represents, we need to link the observed resistance phenotypes with their underlying genomic mechanisms. Broiler chickens represent a key reservoir and vector for NTS infections, but isolates from this setting have been characterized in only very low numbers relative to clinical isolates. In this study, we sequenced and assembled 97 genomes encompassing 7 serotypes isolated from broiler chicken in farms in British Columbia between 2005 and 2008. Through application of machine learning (ML) models to predict the observed AMR phenotype from this genomic data, we were able to generate highly (0.92 to 0.99) precise logistic regression models using known AMR gene annotations as features for 7 antibiotics (amoxicillin-clavulanic acid, ampicillin, cefoxitin, ceftiofur, ceftriaxone, streptomycin, and tetracycline). Similarly, we also trained “reference-free” k-mer-based set-covering machine phenotypic prediction models (0.91 to 1.0 precision) for these antibiotics. By combining the inferred k-mers and logistic regression weights, we identified the primary drivers of AMR for the 7 studied antibiotics in these isolates. With our research representing one of the largest studies of a diverse set of NTS isolates from broiler chicken, we can thus confirm that the *AmpC*-like *CMY-2* β-lactamase is a primary driver of β-lactam resistance and that the phosphotransferases *APH(6)-Id* and *APH(3″-Ib)* are the principal drivers of streptomycin resistance in this important ecosystem.

**IMPORTANCE** Antimicrobial resistance (AMR) represents an existential threat to the function of modern medicine. Genomics and machine learning methods are being increasingly used to analyze and predict AMR. This type of surveillance is very important to try to reduce the impact of AMR. Machine learning models are typically trained using genomic data, but the aspects of the genomes that they use to make predictions are rarely analyzed. In this work, we showed how, by using different types of machine learning models and performing this analysis, it is possible to identify the key genes underlying AMR in nontyphoidal *Salmonella* (NTS). NTS is among the leading cause of foodborne illness globally; however, AMR in NTS has not been heavily studied within the food chain itself. Therefore, in this work we performed a broad-scale analysis of the AMR in NTS isolates from commercial chicken farms and identified some priority AMR genes for surveillance.

## Abstract (precis)

Antimicrobial resistance (AMR) represents an existential threat to the function of modern medicine. Genomics and machine learning methods are being increasingly used to analyze and predict AMR. This type of surveillance is very important to try to reduce the impact of AMR. Machine learning models are typically trained using genomic data, but the aspects of the genomes that they use to make predictions are rarely analyzed. In this work, we showed how, by using different types of machine learning models and performing this analysis, it is possible to identify the key genes underlying AMR in nontyphoidal *Salmonella* (NTS). NTS is among the leading cause of foodborne illness globally; however, AMR in NTS has not been heavily studied within the food chain itself. Therefore, in this work we performed a broad-scale analysis of the AMR in NTS isolates from commercial chicken farms and identified some priority AMR genes for surveillance.

## INTRODUCTION

Genomic methods are being increasingly established as key tools in rapid continuous surveillance, tracking, and control strategy development for infectious diseases (1). They are critical to our ability to study the evolution and spread of antimicrobial resistance (AMR), especially as we adopt a broader One Health (2) approach that integrates clinical, food production, and environmental settings. AMR is a current and growing global health crisis with soaring levels of observed multidrug resistance in a broad range of pathogens (3) combined with record low levels of novel drug discovery (4). There is a global consensus that AMR poses a severe and growing threat to human and animal health (3).

Currently, phenotypic antibiotic susceptibility testing (AST) is the principal method for the identification of AMR in treatment protocol determination and surveillance programs. Unfortunately, AST is highly variable between laboratories and can take days to weeks longer than genomic approaches (5). Despite the development of high-quality curated databases such as the Comprehensive Antibiotic Resistance Database (CARD) (6), we still observe a high level of variability in our ability to predict the phenotypic AMR profile from purely genomic data (7, 8). This disconnect can be attributed to fundamental limitations in the genomic methods used to describe phenotype (i.e., representing genetic capacity but not necessarily gene expression) as well as gaps in our knowledge of resistance determinants. Therefore, despite these expression-related limitations, AST prediction from genomics data is still a highly useful tool for the identification of novel mechanisms and key resistance drivers as well as for determination of the propensity for a given driver to be transmitted. This is vital for prioritizing research and surveillance efforts for the determinants driving AMR.

There have been several approaches used for predicting AST from genomic data sets; these can be divided into AMR gene-centered and gene-free k-mer-based models. The simplest of the approaches in the first category is that of annotation of known AMR genes within the genome and the direct tallying of their associated resistances; for example, in cases in which the genome contained a broad-spectrum β-lactamase such as New Delhi metallo-β-lactamase 1 (NDM-1), the isolate would be considered resistant to β-lactam antibiotics (7, 9). Alternatively, the presence and absence of AMR genes can be used as features to train machine learning (ML) classification models (10). These models learn to determine the weights across the genes that best explain the observed pattern of resistance to a given antibiotic. Such approaches are likely to perform best when organisms are well studied and the AMR mechanisms are relatively well characterized.

The approaches classified into the second category, consisting of the gene-free k-mer-based models, provide an alternative approach. This approach, while more data intensive, attempts to identify the parts of the genome that correlate best to the resistance pattern (11). Gene-free approaches do not include *a priori* assumptions about the AMR determinants in the genome and allow discovery of new genomic features (11–13). However, in certain data sets with limited diversity, such approaches may identify non-AMR-related k-mers that are shared by the resistant organisms only incidentally.

*Salmonella* is a broadly distributed Gram-negative bacterium found in a range of environments throughout the food chain, including in many prominent food-producing animals, e.g., poultry, pigs, and cattle (14). Additionally, it is known to have numerous inter- and intraspecific and environmental transmission routes (15). Nontyphoidal *Salmonella* (NTS) serovars represent the leading global cause of foodborne-related lost years of life (2.4 to 6.2 million years) and lost disability-adjusted life years (2.5 to 6.3 million) (15). Additionally, NTS serovars are conservatively (16) estimated to cause 31.8 to 211.2 million infections globally and 36.3 to 89.1 million deaths annually (15). An increased prevalence of resistant NTS is of great concern due to the association of AMR with worse clinical outcomes (17, 18). Therefore, due to the heavy global burden of NTS, understanding AMR dynamics in this system is highly important.

Previous studies have investigated the utility of genomic methods for AMR prediction of *Salmonella* infections by certain clinical isolates and serovars (7, 19). However, isolates of *Salmonella* from one of the most important vectors of foodborne salmonellosis, chicken, have been previously characterized genomically in only relatively low numbers (20). Therefore, this study aims both to expand this AMR genomic characterization to a broader sampling of NTS serovars isolated from commercial chicken farms and to train prediction models for AMR phenotypes. These models are then used to identify the most important drivers of the observed resistance patterns in this ecological context.

## RESULTS

### Assembly and annotation.

All 97 *de novo* assemblies resulted in genomes comprising between 19 and 105 contigs (mean = 43.56) of average length of between 45.72 kb and 247.03 kb (mean = 122.18 kb). The distribution of *N*_50_ values ranged from a minimum of 68.01 kb to a maximum of 735.38 kb (mean = 341.91 kb), and total genome sizes ranged from 4.64 Mb to 5.05 Mb (mean = 4.84 Mb). G+C% content displayed a tight range over the assembled genomes, with a mean of 52.11% and a standard deviation of 0.11%. All assemblies were deposited in GenBank, and all accession numbers and assembly metrics can be found in Table S1 in the supplemental material.

10.1128/mSystems.00211-19.6 TABLE S1 NCBI GenBank accession numbers and assembly metrics for all genomes (BioProject identifier PRJNA521409). Download Table S1, CSV file, 0.01 MB. © Crown copyright 2019. 2019 Crown This content is distributed under the terms of the Creative Commons Attribution 4.0 International license.

### Phylogeny.

**FIG 1:**
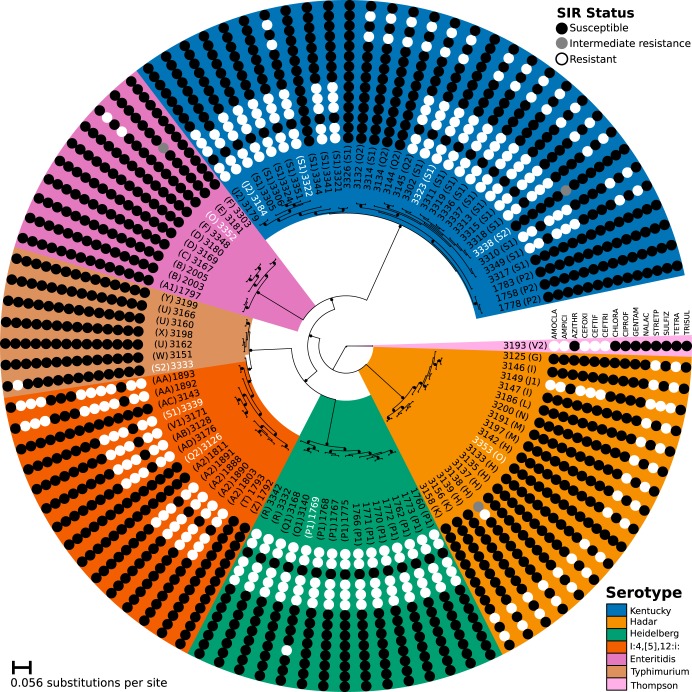
Core genome SNP phylogeny. The figure shows the IQTree maximum likelihood phylogeny generated from core genome SNP alignment. Internal tree nodes with ≥90% ultrafast bootstrap support are noted by black circles. Correspondences of serotype clades to the lowest common ancestor of each are highlighted according to the following color scheme (as indicated by the legend): a blue background indicates *S.* Kentucky serovars, orange *S.* Hadar, green *S.* Heidelberg, red-orange *S.* I:4,[5],12:i:, purple *S.* Enteritidis, brown *S.* Typhimurium, and light pink *S.* Thompson (outgroup). A randomly chosen name representing the farm from which a sample was isolated is indicated in parentheses. AST results are indicated using circles, with resistance indicated by a white circle, intermediate resistance by a gray circle, and susceptibility by a black circle. Antibiotics are abbreviated per standard shorthand from the taxon label outward as follows: amoxicillin-clavulanic acid (AMOCLA), ampicillin (AMPICI), azithromycin (AZITHR), cefoxitin (CEFOXI), ceftiofur (CEFTIF), ceftriaxone (CEFTRI), chloramphenicol (CHLORA), ciprofloxacin (CIPROF), gentamicin (GENTAM), nalidixic acid (NALAC), streptomycin (STREPT), sulfamethoxazole (SULFIZ), tetracycline (TETRA), and trimethoprim-sulfamethoxazole (TRISUL). Taxa for which the AST was systematically predicted incorrectly are indicated in white characters.

Phylogenetic analysis of a core genome single nucleotide polymorphism (SNP) alignment shows a relatively well-supported monophyletic distribution of serotypes (see Fig. 1). Isolate 3333 was the one exception to this monophyly, branching with 100% ultrafast bootstrap support as sister to the well (100%)-supported Salmonella enterica serovar *S.* I:4,[5],12:i: clade.

### Antibiotic susceptibility testing.

In contrast to the largely monophyletic serotype patterns observed in the phylogenetic analysis, the observed complements of resistances to the antibiotics tested showed variation both within and between the serovars (Fig. 1; see also Table S2). *S.* Kentucky serotypes, in particular, displayed the greatest range of observed AMR phenotypes, with 6 distinct patterns of resistance. *S.* Hadar serotypes had 4 resistance sets, and *S.* I:4,[5],12:i: serotypes displayed 3 different sets. The remaining isolates with more than a single exemplar, namely, *S.* Typhimurium, *S.* Enteritidis, and *S.* Heidelberg, were more consistent, showing only 2 different resistance patterns each.

10.1128/mSystems.00211-19.7 TABLE S2 Full MIC results for all isolates and their CLSI breakpoint SIR status. Download Table S2, XLSX file, 0.01 MB. © Crown copyright 2019. 2019 Crown This content is distributed under the terms of the Creative Commons Attribution 4.0 International license.

The most commonly observed set of cooccurring resistances (see Fig. S1G in the supplemental material) to a group of antibiotics consisted of the 48.4% (47/97) of isolates resistant to all the β-lactam antibiotics tested, i.e., the aminopenicillins (amoxicillin-clavulanic acid [AMOCLA] and ampicillin [AMPICI]) and the cephalosporins (cefoxitin [CEFOXI], ceftiofur [CEFTIF], and ceftriaxone [CEFTRI]). Only 2 isolates were susceptible to some but not all β-lactam antibiotics: a single *S.* Kentucky isolate (3184) that was susceptible to CEFTRI while being resistant to the other β-lactams and a single *S.* Hadar isolate (3138) that showed intermediate resistance and full resistance to AMOCLA and AMPICI, respectively, but susceptibility to the 3 cephalosporins. In terms of serotypes, this group of β-lactam-resistant isolates included all of the *S.* Heidelberg isolates, 20/32 (65.63%) of the *S.* Kentucky isolates, 10/15 (66.6%) of the *S.* I:4,[5],12:i: isolates, 1/17 (5.9%) of the *S.* Hadar isolates, and the lone *S.* Thompson isolate.

10.1128/mSystems.00211-19.1 FIG S1 (A to G) Observed number of resistant isolates by serotype. Antimicrobials are presented in the same order for all panels as indicated by axis label on the bottommost panels. (H) Cooccurrence pattern of resistance across all serotypes. This UpSet plot shows the different sets of resistances observed across the isolates. The top bar plot indicates how many isolates were resistant to each specific group of antibiotics. The corresponding set of antibiotics is indicated below the top bar plot by the dark colored dots. The bar plot to the left shows how frequently resistance to each antibiotic occurred regardless of which set of resistances it appeared within. Download FIG S1, PDF file, 0.1 MB. © Crown copyright 2019. 2019 Crown This content is distributed under the terms of the Creative Commons Attribution 4.0 International license.

The other most commonly observed group of shared resistances (see Fig. S1H) was that consisting of the 36.1% (35/97) of isolates resistant to the aminoglycoside streptomycin (STREPT) and tetracycline (TETRA). This pattern of shared resistances included 16/17 (94.1%) of the *S.* Hadar isolates, 16/32 (50%) of the *S.* Kentucky isolates, 2/15 (13.3%) of the *S.* I:4,[5],12:i: isolates, and 1/10 (10%) of the *S.* Enteritidis isolates. There were only 2 exceptions to the pattern of isolates being resistant to either of STREPT or TETRA (implying resistance to the other): *S.* Typhimurium isolate 3333 and *S.* Heidelberg isolate 1769.

Constituting a set apart from those resistant to the β-lactams, streptomycin, and tetracycline, only 5 isolates were resistant to any other antibiotic tested: 2/15 (13.3%) of the *S.* I:4,[5],12:i: isolates (1892 and 1893) showed resistance to chloramphenicol (CHLORA) and sulfamethoxazole (SULFIZ), a single (1/32, 3.13%) *S.* Kentucky isolate (3338) and a single (1/10, 10%) *S.* Enteritidis isolate (3181) had intermediate resistance to CHLORA (see Fig. S2), and, finally, a single *S.* Hadar isolate (1/17 5.9%) was also resistant to SULFIZ.

10.1128/mSystems.00211-19.2 FIG S2 Hierarchical clustering of observed phenotypic AST results. Black cells indicate susceptibility, while red and cream cells represent intermediate susceptibility and resistance, respectively. The serotypes are indicated along the left axis per the legend. Each row represents a single isolate and each column a single antibiotic tested. Isolates and antibiotics are ordered according to their similarity to one another as inferred by a pair of hierarchical clustering inferences as shown by the pair of annotated dendrograms. The antibiotics tested were amoxicillin-clavulanic acid (AMOCLA), ampicillin (AMPICI), azithromycin (AZITHR), cefoxitin (CEFOXI), ceftiofur (CEFTIF), ceftriaxone (CEFTRI), chloramphenicol (CHLORA), ciprofloxacin (CIPROF), gentamicin (GENTAM), nalidixic acid (NALAC), streptomycin (STREPT), sulfamethoxazole (SULFIZ), tetracycline (TETRA), and trimethoprim-sulfamethoxazole (TRISUL). Download FIG S2, PDF file, 0.04 MB. © Crown copyright 2019. 2019 Crown This content is distributed under the terms of the Creative Commons Attribution 4.0 International license.

The isolates showing resistance to the greatest numbers of antibiotics (i.e., the most multiresistant isolates) were *S.* I:4,[5],12:i: isolates (1893 and 1892) and the *S.* Hadar isolate (3149), which were resistant to chloramphenicol and sulfamethoxazole, respectively, as well as to the β-lactams and to the aminoglycoside and tetracycline tested. A total of 12/32 (37.5%) *S.* Kentucky isolates were resistant to all of these antibiotics apart from chloramphenicol and sulfamethoxazole. At the other end of the spectrum, *S.* Enteritidis was the most susceptible serotype with 9/10 (90%) of isolates showing susceptibility (or intermediate susceptibility) to all antibiotics. Similarly, 6/7 (85.7%) of the *S.* Typhimurium isolates, 7/32 (21.88%) of the *S.* Kentucky isolates, 5/15 (33.3%) of the *S.* I:4,[5],12:i: isolates, and 1/17 (5.9%) of the *S.* Hadar isolates also showed susceptibility (or intermediate susceptibility) to all tested antibiotics.

### Antimicrobial resistance gene analysis.

**FIG 2:**
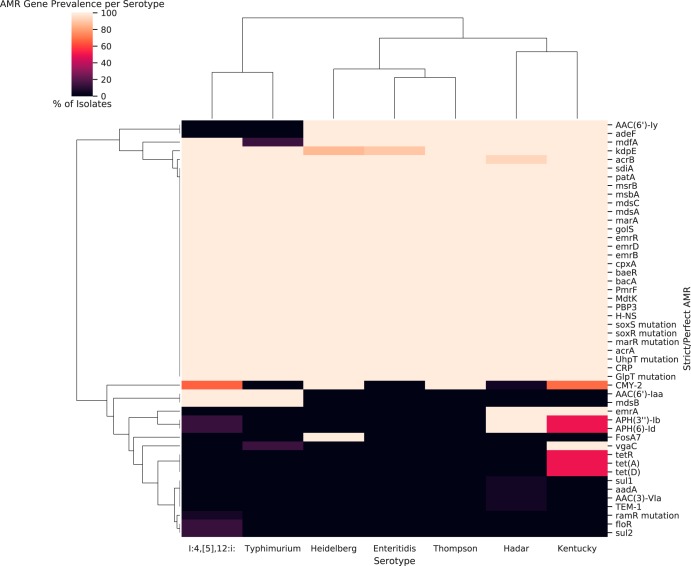
All AMR genes detected by serotype under CARD’s “Strict” and “Perfect” criteria (including efflux system components). Each cell indicates the percentage of isolates belonging to the relevant serotype (column) that contained each detected determinant (rows). Black blocks indicate that no isolates of that serotype had that AMR gene, and cream blocks indicate that 100% of the isolates had that AMR gene. Serotypes and genes are each ordered via hierarchical clustering as indicated by the dendrograms.

Twenty-five putative AMR genes were identified across all isolates and serotypes by CARD’s “strict” or “perfect” match criteria (see Fig. 2). Every isolate had a pair of fosfomycin resistance-related mutations in the *glpT* and *uhpT* transporters (21) as well as a *pmrF* gene associated with resistance to polymyxin antibiotics (22), a Penicillin-binding protein (PBP) 3 gene (23), and a *bacA* gene (associated with low-level resistance to bacitracin) (24). Additionally, there were 16 efflux components and determinants associated with their expression, including general efflux regulators such as H-NS histone-like protein (25) and *cpxA* (26). Genes implicated in specific efflux systems included genes associated with the *acrAB* system: *sdiA* (27), *marR* and *marA* (28), and *soxR* and *soxS* (29). Interestingly, while *acrB* was detected in all but a single *S.* Hadar strain, *acrA* was not detected in any isolate. The other ubiquitous efflux pump-related systems included *mdsABC*-associated genes *golS*, *mdsA*, and *mdsC* (30); MATE efflux system *mdt*-associated efflux components *CRP* (31), *baeR* (32), and *mdtK* (33); and *emrAB-TolC* components and regulators *emrB*, *emrD*, and *emrR* (34, 35). Interestingly, only the *S.* Hadar and *S.* Kentucky isolates had *emrA* and only the *S.* Typhimurium and *S.* I:4,[5],12:i: isolates had *mdsB*. Finally, there were *patA* and *msbA* transporters found in all isolates despite no detection of *patB* (36). *kdpE*, a regulator of potassium transport associated with aminoglycoside resistance (37), was nearly ubiquitous (absent in 2/15 *S.* Heidelberg and 1/10 *S.* Enteritidis isolates).

Some predicted AMR genes had more varied serotype distributions. The chloramphenicol exporter *mdfA* (38) was ubiquitous in all serotypes apart from *S.* Typhimurium, where it was present in a single isolate (1/7). Similarly, the aminoglycoside resistance gene *AAC(6’)-Iy* (39) and the *adeFGH* efflux component *adeF* (40) were present in all genomes apart from those of the *S.* Typhimurium and *S.* I:4,[5],12:i: isolates. The β-lactamase *CMY-2* gene (41) was present in all of the *S.* Heidelberg and *S.* Thompson strains but absent from the *S.* Enteritidis and *S.* Typhimurium isolates. *CMY-2* was more unevenly distributed in other serotypes, being detected in 5.88% of *S.* Hadar isolates, 66.67% of I:4,[5],12:I isolates, and 68.75% of *S.* Kentucky isolates. All *S.* Typhimurium and *S.* I:4,[5],12:i: isolates but no isolates of other serotypes contained *AAC(6’)-Iaa*. Similarly, *APH(6)-Id* was found in all *S.* Hadar isolates but in only 13.33% of the *S.* I:4,[5],12:i: isolates and 50% of the *S.* Kentucky isolates. Every *S.* Kentucky isolate also had the streptogramin A resistance-related *vgaC* gene (42), but it was otherwise found only in a single *S.* Typhimurium isolate (3333).

AMR genes detected within only a single serotype included *fosA7* in every *S.* Heidelberg isolate sequenced, and *tet* genes corresponding to efflux pump-related proteins, namely, *tetR*, *tetD*, and *tetA* (43), were found exclusively in 16/32 (50%) of *S.* Kentucky isolates. The rarest AMR genes in our data set, *floR* (phenicol resistance) (44) and *sul2* (sulfonamide resistance) (45), were found in only 2 (13.3%) of *S.* I:4,[5],12:i: isolates (1892 and 1893) collected from the same farm. Another *S.* I:4,[5],12:i: isolate was the lone carrier of *ramR* mutations related to upregulation of *acrAB* (46). Finally, the *TEM-1* β-lactamase gene (47) was found in only a single *S.* Hadar isolate (3142), with *aadA*, *sul1*, and *AAC(3)-VIa* found in only a single different *S.* Hadar isolate (3186).

### Predicting phenotype from genotype.

The relationship between the observed AST phenotype and the AMR determinants detected within the sequenced genomes was assessed according to standard FDA categories. If the predictions from the genome or models trained using the genome matched the phenotypic data, then the result was classified as representing “categorical agreement.” However, if the prediction was of resistance but the phenotype showed susceptibility, then the result was classified as representing a “major disagreement,” and if the prediction was of susceptibility but the phenotype showed resistance, then the result was classified as representing a “very major disagreement.”

**FIG 3:**
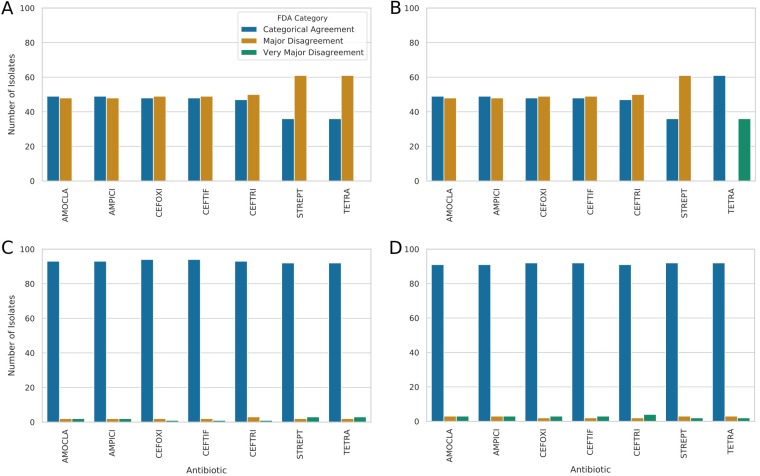
FDA categorization of AST prediction performance across the antibiotics with sufficient numbers of susceptible and resistant isolates for assessment. “Categorical Agreement” represents the cases in which the prediction matched the observed phenotype, “Major Disagreement” corresponds to a prediction of resistance but a determination of susceptibility by the AST, and “Very Major Disagreement” indicates a prediction of susceptibility but a determination of resistance by the AST. (A) Performance of direct tallying of the presence of AMR genes as detected by RGI. (B) The same procedure was performed but with exclusion of efflux determinants. (C) Accuracy of prediction of resistance patterns by the use of binary logistic regression models trained using the AMR genes as features. (D) Accuracy of prediction of resistance directly from the genome by the use of a k-mer-based set-covering machine model.

**FIG 4:**
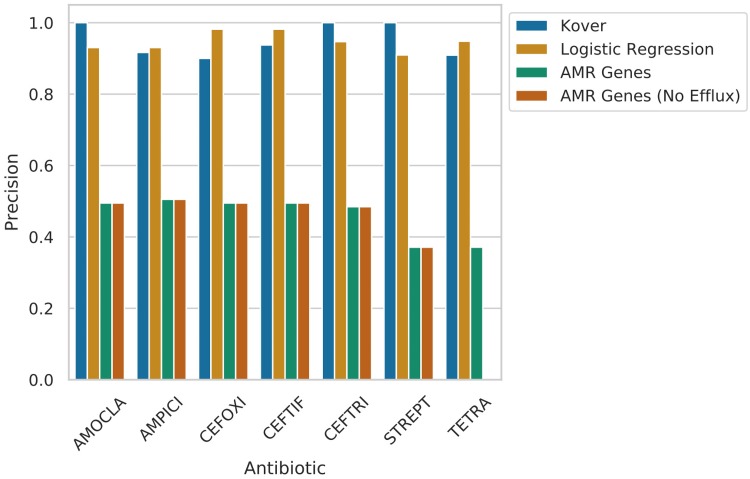
Observed precision for direct tallying with and without efflux pumps and test set average classifier precision for set-covering machine and logistic regression models. These results clearly show that both machine learning approaches created far more precise predictions of AST (>0.9) than direct tallying of the AMR determinants.

By using the antibiotic resistance ontology (ARO) that CARD is built upon (48), we were able to directly tally the associated resistances with the detected AMR determinants. As can be seen in the high levels of major disagreement (48% to 62% of isolates; Fig. 3) and poor precision (0 to 0.5; Fig. 4), directly tallying resulted in a massive overprediction of resistance. As this was initially believed to be due to the presence of the efflux pump components, we also performed this direct tallying without efflux pumps but observed the same overprediction of resistance. The one exception to this pattern was tetracycline resistance, which was consistently underpredicted in the absence of efflux (i.e., all isolates were classified as “susceptible with very major disagreement”). Overall, the level of precision seen from direct tallying of ranges is low, at approximately 0.5 for β-lactams and 0.38 for streptomycin and tetracycline (0.0 precision with no efflux genes for tetracycline). This represents the underlying proportion of resistant isolates in our data set.

### Logistic regression.

A simple set of binary logistic regression models using detected AMR genes as features were able to predict the AST. On a held-out test set, average precision ranged from 0.91 for streptomycin to 0.98 for ceftiofur and cefoxitin (see Fig. 4). This meant that, overall, there were a maximum of only 3 major disagreements and a maximum of 4 very major disagreements among the 97 isolates.

**FIG 5:**
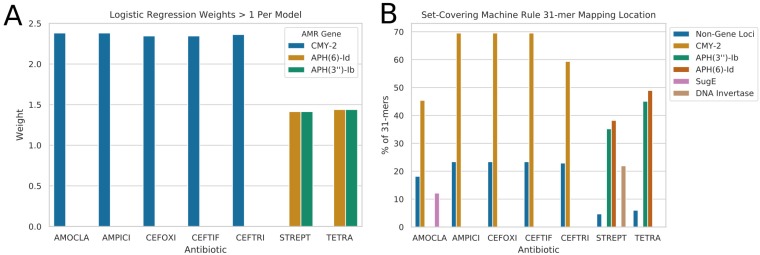
A plot of the most important features and their identity for the machine learning models. (A) Learnt coefficients/weights on the AMR gene presence/absence matrix by the logistic regression models. Only weights greater than 1 are displayed on this plot. (B) Top genomic origins of the 31-mers learnt by the set-covering machines. Non-Gene Loci are the 31-mers mapping outside any of the identified genes.

Inspection of the mostly highly weighted AMR determinants for each trained logistic regression model revealed that the β-lactamase *AmpC*-like *CMY-2* gene was most important for prediction of resistance to β-lactam antibiotics (see Fig. 5). No other determinant had a weighting of greater than 25% of that of *CMY-2* for the β-lactam models. For the streptomycin and tetracycline models, the phosphotransferase genes *APH(6)-Id* and *APH(3”)-Ib* were the most highly weighted determinants by a factor of 2 or greater.

### Set-covering machine.

Similarly, the k-mer-based set-covering machine classifiers also greatly outperformed direct tallying. Set-covering machines (implemented using the Kover algorithm as described previously [13, 49]) represent a type of machine learning model that learns a set of Boolean rules, e.g., presence/absence (and higher-order conjunctions) of specific features (k-mers in our case) which predict the resistance label (50).

Performance for all 7 antibiotics represented greater than 0.9 precision and was only slightly poorer than that of logistic regression despite having only the genomic 31-mers as features (see Fig. 3; see also Fig. 4). Overall, under the FDA categorization metrics, the set-covering machines performed similarly to logistic regression and considerably better than direct tallying.

When the 31-mers identified by the trained set-covering machines as the most highly equivalently important rules to predict AST were mapped back to the underlying genomes, the majority mapped to the same AMR genes that were most highly weighted by logistic regression, i.e., *CMY-2* for β-lactam antibiotics and *APH(3”)-Ib* and *APH(6)-Id* for streptomycin and tetracycline (see Fig. 5). However, there were a few additionally weighted non-AMR-specific genes that had significant numbers of mapping k-mers. For example, the amoxicillin-clavulanic acid model included 12.1% of k-mers mapping to the gene *sugE*, and the streptomycin model had a reasonably high 21.9% rate of mapping to a *hin* DNA invertase gene.

## DISCUSSION

Overall, the serotypes isolated from broiler chicken in this study included the top 5 serovars implicated in human salmonellosis in Canada (51). The most common clinical isolate serovar, *S.* Enteritidis (56% of cases) (51), was relatively less common in the chicken isolates studied here (10.31%) than in the clinical data. This might reflect the earlier sampling date of these isolates, as the most recent Canadian FoodNet report indicated that 41% of *Salmonella* isolates from broiler chicken manure at sentinel sites were *S.* Enteritidis (51). As *S.* Enteritidis has become more common in Canadian salmonellosis cases, it is perhaps reassuring that all broiler chicken *S.* Enteritidis isolates in this study were totally susceptible to all 14 tested antibiotics. Other serotypes either were found in our isolates in proportions similar to those of the Canadian clinical isolates (for *S.* Typhimurium, 7.2% versus 7%) or were more common in our data set, including *S.* Heidelberg (31.9% versus 3%) and *S.* I:4,[5],12:i: (15.4% versus 3%) (51). Rare clinical isolates such as *S.* Kentucky (33%) and *S.* Hadar (17.5%) were far more common in our isolates, but *S.* Infantis, which is the 5th most common cause of salmonellosis (3%) (51), was totally absent. These observed overlaps and differences with respect to the diversity of human clinical and broiler chicken NTS isolates underscore the utility and relevance of this type of genomic surveillance work.

The *Salmonella in silico* typing resource (SISTR) in-silico serotyping conducted is supported by the largely monophyletic distribution of serotypes within the core genome phylogeny (Fig. 1). There was only one exception to this pattern, namely, an *S.* Typhimurium isolate (3333) which branched within the I:4,[5]:12:i clade. As this is a monophasic *S.* Typhimurium variant, we would expect this to branch within the phylogenetically adjacent *S.* Typhimurium clade. This suggests that this (well-supported) branching location might reflect some form of phylogenetic reconstruction artifact. Otherwise, the inferred relationships between serotype clades largely agree with those previously inferred in dedicated *Salmonella* phylogenomic analyses (52).

There were limitations to this data set for the purposes of predicting phenotypic resistance from genomic data. Tests of several antibiotics identified no or very few resistant isolates, meaning that it was possible to train models for only a subset of antibiotics (e.g., AMOCLA, AMPICI, CEFOXI, CEFTIF, CEFTRI, STREPT, and TETRA) due to the problem of label imbalance. The treatment of phenotypes showing intermediate resistance as resistant is another potential cause of distortion in the trained models. However, only a single model (AMOCLA) used any isolates with intermediate phenotypes (a single isolate, representing 1.03% of the data). This means that, even if it were misleading to treat this isolate as resistant, doing so would have a correspondingly small effect on the learnt weights and k-mer mappings of the AMOCLA models. Finally, we do lose potentially interesting granularity in our models by performing only binary predictions of susceptibility or resistance. Ideally, we would train regression models that directly predict MIC values. Unfortunately, this is a difficult problem due to this data set being somewhat small for this level of prediction and due to the nature of MIC measurement. MICs are generally measured to within an accuracy of only a 2-fold dilution, meaning that the amount of measurement error is greater for higher MIC values than for lower ones. The other problem is that MICs at the extremes of the standardized measured range are presented only as inequalities (i.e., >256 mg/liter or <0.5 mg/liter) which is difficult to handle mathematically. However, future work incorporating larger numbers of genomes and measurements could use approaches such as maximum margin interval trees to address these issues (53).

This being said, by using the learnt weights and k-mer mapping locations (see Fig. 5) in the high-precision (see Fig. 4) AMR gene-based and gene-free resistance prediction models generated in this study, it was possible to identify key drivers of observed resistance patterns for the subset of antibiotics with a reasonable balance of resistant and susceptible isolates in our data set. Therefore, we attempted to identify key AMR drivers for β-lactam resistance (specifically, amoxicillin-clavulanic acid, ampicillin, cefoxitin, ceftiofur, and ceftriaxone) as well as for streptomycin resistance and tetracycline resistance. It should be noted that these results are specific to the isolates and serovars within our data set; while there are some highly encouraging functional connections, expanding this approach to incorporate new serotypes would be best served by retraining the models.

The β-lactam models were largely identical, pinpointing the β-lactamase *CMY-2* gene (or k-mers derived from this gene) as the most important feature in all individual models. Only the amoxicillin-clavulanic acid k-mer model featured less than 50% k-mers deriving from a *CMY-2* gene. This model featured a low but notable proportion of k-mers (12.1%) deriving from a *sugE* gene. *sugE* is associated with resistance to quaternary ammonium compounds (forming part of an efflux pump), specifically, cetylpyridinium, cetyldimethylethyl ammonium, and cetrimide, cations commonly used as disinfectants (54). This gene has previously been detected on the same plasmid as *CMY-2* (55) and may play a coselection role. Every single isolate with *CMY-2*, with the exception of 3186, had a directly adjacent lipocalin gene (*blc*) on the same strand followed by a *sugE* gene in the opposite orientation (see Fig. S4 in the supplemental material). This suggests that *sugE* was likely learnt to be predictive purely due to its colocation/linkage instead of due to any specific antimicrobial resistance-related function. The presence of this adjacent *blc* may further potentiate the resistance, as these have been reported in other bacteria to increase MICs of β-lactam antibiotics by binding the antibiotic in the medium (56, 57).

This result is supported by previous work identifying *CMY-2* as a key driver of extended-spectrum-β-lactam resistance in Escherichia coli derived from broiler chickens (58). *CMY-2* has been established as displaying relatively broad distribution in samples derived from a range of bacteria and food production animals in Canada (59) as well as globally (60–62). Importantly, evidence of the direct connections of NTS with *CMY-2*-related extended-spectrum-β-lactam resistance in human clinical infections has been established (if somewhat poorly understood) (63). Previous work in poultry E. coli isolates has shown that without active selection from antibiotic usage, this gene is rapidly lost in chicken samples (58). However, *CMY-2* does persist in poultry farm environmental samples (58), suggesting that further work is needed to elucidate the selective forces determining persistence and transmission of this critical resistance gene. One potential avenue for this work, underlined by the k-mers derived from the *sugE* gene, is that of looking at the role which the use of quaternary ammonium-based disinfectants plays in this process. Similarly, there is a need to experimentally investigate whether the presence of *blc* can lead to increased β-lactam resistance in *Salmonella* such as they have been shown to do in other bacteria (56, 57) and what impact this has on the retention of *CMY-2*.

For both the logistic regression and k-mer set-covering machine models, there were a subset of genomes that were consistently mispredicted (whether they were in the training set or the test set). Specifically, for the β-lactam antimicrobials (AMPICI, AMOCLA, CEFOXI, CEFTRI, and CEFTIF), every individual model failed to correctly predict the phenotype for isolates 3338, 3126, and 3339, with one additional misprediction in just the CEFTRI models for 3184. These taxa are highlighted in Fig. 1 with white taxon names and do not exhibit any clear monophyletic phylogenetic groupings, serotype-based trend, or sampling location. It should be noted, however, that these taxa displayed resistance phenotypes that were different from those of their closest relatives despite similar predicted AMR gene complements; e.g., 3333 is the only *S.* Typhimurium isolate with any observed resistance, and 3352 is the only *S.* Enteritidis isolate with clear resistance phenotypes.

Some isolates that were predicted to be resistant to β-lactams but were found to be susceptible in the AST showed a perfect hit for the *CMY-2* β-lactamase (see Fig. 2). Specifically, isolates 3186, 3126, and 3338 all had *CMY-2* genes clearly present but were found to be susceptible to β-lactams, with the observed MICs at the lower end of the measured range (see Table S2 in the supplemental material). These inferred susceptibilities were consistent even following multiple replications of the AST results. This likely suggests an uncharacterized context-related expression determinant for *CMY-2* (i.e., the gene is present but not expressed) to explain why these strains had the capacity for β-lactam resistance but did not display that phenotype during testing. This could be tested in future work via approaches that directly measure the presence of specific proteins such as matrix-assisted laser desorption ionization–time of flight (MALDI-TOF) mass spectrometry (64). On the other hand, *S.* I:4,[5],12:i isolate 3339 and *S.* Hadar isolate 3149 were both resistant to the β-lactam tested but had no detectable *CMY-2* gene. Additionally, with the exception of genes of efflux pump components, there were no AMR genes or mutations clearly associated with β-lactam resistance detected in these isolates. However, previous work has shown that *Salmonella* can display resistance to β-lactam antibiotics without any detectable β-lactamase genes being present (65). This suggests the presence of an undetected or uncharacterized β-lactam resistance mechanism in these isolates. It is possible that the 18% to 23.3% of k-mers mapping to intergenic regions for these models may play a role in this resistance mechanism.

For the streptomycin resistance models, the most important predictor was the presence of the phosphotransferase genes *APH(6)-Id* and *APH(3”)-Ib*. These are two among a large number of known aminoglycoside resistance genes detected in *Salmonella* isolates (66). They are also frequently found on mobile genetic elements such as transposons and plasmids (66). Additionally, they have previously been associated with resistance to streptomycin in animal isolates (67). Interestingly, there were also a number of k-mers (21.9%) associated with a *hin* DNA invertase gene in the set-covering machine model for streptomycin. This gene has been previously associated with phase variation in *Salmonella* (68).

Similarly to the β-lactam models, there was a subset of isolates what were consistently mispredicted for streptomycin (3322, 3323, 3352, 3353, and 1769). Among the isolates where there was a failure to successfully predict streptomycin resistance, it was found that these had no detectable *APH(6)-Id* or *APH(3″)-Ib* genes. However, several did show evidence of a cryptic aminoglycoside N-acetyltransferase enzyme gene, *ACC(6′)-Iy*, in their genome (isolates 3322, 3352, and 1769) but so did a large number of isolates that displayed susceptibility to streptomycin (e.g., every *S.* Enteritidis, *S.* Heidelberg, *S.* Thompson, *S.* Hadar, and *S.* Kentucky isolate). All 3 of the isolates with the cryptic aminoglycoside N-acetyltransferase enzyme gene *ACC(6′)-Iy* were the lone examples of a resistant isolate within clades that otherwise contained only susceptible isolates. In the other direction, isolate 3323 was predicted to be resistant due to the presence of both the *APH(6)-Id* gene and the *APH(3″)-Ib* gene but was found to be susceptible to streptomycin in the AST. Similarly, this isolate branched within a resistant clade that was found to be totally resistant to these antibiotics. Isolate 3353 displayed a pattern similar to that shown by the lone *S.* Hadar isolate and thus was found not to be resistant to streptomycin despite having a strict full-length hit with respect to *APH(6)-Id* and 100% identity and a partial (52%) hit to *APH(3″)-Ib*. These prediction failures were not attributable to a failure to detect AMR genes due to poor assembly quality. All the consistently mispredicted genomes had assembly quality metrics that were largely representative of the assemblies overall (see Fig. S3).

10.1128/mSystems.00211-19.3 FIG S3 Visualization of the *N*_50_ distribution of assembled genomes. This figure shows that the assemblies of genomes that were consistently mispredicted in terms of AMR for TETRA/STREPT and the β-lactam antibiotics tested are not notably more fragmented than the assemblies as a whole. Download FIG S3, PDF file, 0.03 MB. © Crown copyright 2019. 2019 Crown This content is distributed under the terms of the Creative Commons Attribution 4.0 International license.

10.1128/mSystems.00211-19.4 FIG S4 *CMY-2*. The *blaCMY-2* genes were typically flanked by *ISEcp1* downstream and lipocalin (*blc*) on the same strand followed by a quaternary ammonium compound(s) (*sugE*) on the opposite strand. Download FIG S4, PDF file, 0.08 MB. © Crown copyright 2019. 2019 Crown This content is distributed under the terms of the Creative Commons Attribution 4.0 International license.

The data corresponding to the tetracycline resistance model weights and k-mer locations were more perplexing. Specifically, the learnt weights and k-mers in that model were nearly identical to those seen with the streptomycin models despite the differences in the drug classes and known resistance mechanisms. These phosphotransferase genes [*APH(6)-Id* and *APH(3″)-Ib*] have never been associated with a mechanism through which they could convey tetracycline resistance directly. This result renders the tetracycline model somewhat suspect and could represent overfitting to the training data due to the strong cooccurrence of streptomycin resistance and tetracycline resistance among isolates in our data set. While these aminoglycoside resistance genes are frequently detected on chromosomal fragments containing the *tet*(*B*) tetracycline resistance gene (69), this particular tetracycline gene was totally absent in these isolates, so there is little evidence to support any hypothesis involving colocalization. In terms of mispredicted isolates, they was largely the same isolates as were seen in the streptomycin testing (3322, 3323, 3352, and 3353), with the exception of 1769 and the addition of isolate 3333.

Overall, this work shows the potential utility and pitfalls of the effective use of genomic data for the surveillance of AMR. We demonstrate the propensity of overprediction of resistance that occurs in tallying resistance directly from AMR gene predictions. Additionally, we show the utility of comparing the learnt parameters of simple machine learning models to help identify key drivers of antimicrobial resistance. Specifically, we identify the *AmpC*-like β-lactamase *CMY-2* gene as the primary driver of resistance to aminopenicillins and to second- and third-generation cephalosporins in broiler chicken nontyphoidal Salmonella enterica serovars (see Fig. 5; see also Table S4). As this β-lactamase has been reported in human NTS isolates (63), this underscores the importance of monitoring of *CMY-2* β-lactamase prevalence and transmission in food production both within Canada (59) and globally (60–62). This work also revealed that *APH(6)-Id* and *APH(3″)-Ib* genes are key determinants driving streptomycin resistance in Canadian chicken NTS isolates (see Fig. 5; see also Table S4). Reassuringly, the most commonly clinically relevant serotype, *S.* Enteritidis, was shown to be susceptible to all common antimicrobials in Canadian poultry sources. Additionally, despite previous detection of colistin resistance genes in *CMY-2*-bearing poultry isolates (70) there was no evidence of colistin resistance genes present in the genomes of these isolates based on the Resistance Gene Identifier (RGI) analyses (see Fig. 2).

## MATERIALS AND METHODS

### Isolation.

A total of 97 *Salmonella* serovar isolates obtained from 23 broiler chicken farms in British Columbia, Canada, were sequenced in this study. Isolates were selected based on their prevalence, pulsotype, and antibiotic susceptibility profiles as outlined previously (71).

### Sequencing.

Genomic DNA was extracted from overnight cultures in 5 ml of brain heart infusion (BHI) broth (BD, NJ, USA) using DNeasy blood and tissue kits (Qiagen) as specified in the protocols (71).

The extracted DNA was stored in 10 mM Tris-HCl buffer (pH 8.0) and quantified by the use of an Invitrogen Qubit 2.0 Fluorometer (Life Technologies). The quality of DNA was visualized by electrophoresis on a 1% agarose gel, and the DNA was stored at −20°C until construction of the genomic libraries. The libraries were then sequenced using a MiSeq v3 sequencer in paired-end mode to generate 2 × 250-bp reads.

### Assembly and annotation.

Genomes were assembled using a standardized MiSeq assembly pipeline implemented within the Integrated Rapid Infectious Disease Analysis (IRIDA) platform of the Public Health Agency of Canada (72). This workflow trims reads to remove low-quality sequences and then merges overlapping paired reads with Fast Length Adjustment of SHort (FLASH) (v1.2.9) reads (73). The merged and remaining unmerged reads were then used to generate a *de novo* assembly with SPAdes (v3.9.0) (74). The resultant assembly was then filtered to remove short (>1,000-bp) contigs, repetitive (1.75 repeat cutoff ratio) contigs, and low-coverage (0.33 coverage cutoff ratio) contigs.

Assembly metrics were calculated for the final assemblies using QUAST (v5.0.2) (75) (see Table S1 in the supplemental material). The final assemblies were annotated with Prokka (v1.13) (76) using the “*Salmonella*” genus, “*enterica*” species, and Gram-negative options and a 1E−5 minimum expectation value. Additionally, assemblies were screened for plasmids using abricate (v0.8.7) (77) and the plasmidfinder database (27 August 2018) (78). Plasmid screening results were then summarized and visualized using the Pandas (v0.22.0) (79) and Seaborn (v0.8.1) Python libraries (80) (see Fig. S5 in the supplemental material; see also Table S3).

10.1128/mSystems.00211-19.5 FIG S5 Visualization of the relative levels of coverage of plasmids within the genome assemblies as detected using abricate and the plasmidfinder database. Download FIG S5, PDF file, 0.04 MB. © Crown copyright 2019. 2019 Crown This content is distributed under the terms of the Creative Commons Attribution 4.0 International license.

10.1128/mSystems.00211-19.8 TABLE S3 Plasmid prediction results from the genomes studied (determined via the plasmidFinder database and abricate). Download Table S3, CSV file, 0.01 MB. © Crown copyright 2019. 2019 Crown This content is distributed under the terms of the Creative Commons Attribution 4.0 International license.

10.1128/mSystems.00211-19.9 TABLE S4 Relative risk calculations (determined using SAS-implemented chi-square tests) for the subset of AMR genes identified in the predictive modeling and literature as key drivers of resistance. Download Table S4, CSV file, 0.01 MB. © Crown copyright 2019. 2019 Crown This content is distributed under the terms of the Creative Commons Attribution 4.0 International license.

### Serotyping.

Serotyping was performed using the assembled genomic contigs and the *Salmonella* In Silico Typing Resource (SISTR) tool (v1.0.2) (81).

Code for this and all further analyses in this paper can be found in the associated Jupyter Notebook (82) and in the relevant folder under “analyses” in the git repository (83) (i.e., in this case, “analyses/serotyping”). This repository can be found at https://github.com/fmaguire/salmonella_ast_prediction/.

### Phylogenetics.

A pangenome analysis was conducted using the Prokka annotations and Roary (v3.12.0) (84). From the identified 3,743 core genes present in ≥99% of genomes, an alignment was inferred in Roary. SNPs were then extracted from this alignment using “snp-sites” (v2.4.0) (85). This resulted in an alignment consisting of 77,795 sites.

A maximum likelihood phylogeny was then inferred in IQTree (v1.6.5) (86, 87) with UltraFast Bootstrap support. The inference was performed under the General Time Reversible Model with ascertainment bias correction and nucleotide frequencies (GTR+ASC+F), as this was the consensus best-fitting model for all information criteria as determined by ModelFinder (88). Phylogeny was then visualized and annotated with SISTR serotyping, AST results, and encoded origin using ETE3 (v3.1.1) (89).

Code used to perform this can be found in the notebook under “analyses/phylogeny.”

### Antibiotic susceptibility testing.

Phenotypic antibiotic susceptibility testing (AST) was conducted for a panel of 14 standard antibiotics as described previously (71). In brief, a Sensititre automated system (Trek Diagnostic Systems, Cleveland, OH) was used to determine MICs (Table S2), and the results were analyzed according to Clinical and Laboratory Standards Institute guidelines for the following antibiotics: amoxicillin-clavulanic acid (AMOCLA), ampicillin (AMPICI), azithromycin (AZITHR), cefoxitin (CEFOXI), ceftiofur (CEFTIF), ceftriaxone (CEFTRI), chloramphenicol (CHLORA), ciprofloxacin (CIPROF), gentamicin (GENTAM), nalidixic acid (NALAC), streptomycin (STREPT), sulfamethoxazole (SULFIZ), tetracycline (TETRA), and trimethoprim-sulfamethoxazole (TRISUL).

This phenotypic testing was fully repeated for all isolates to confirm the resistance status. The code used to visualize and explore these results is available in the Jupyter Notebook under “analyses/ast.”

### AMR gene identification.

AMR gene identification was performed using Resistance Gene Identifier (RGI) v4.0.3 (6) on assembled contigs. This involved Prodigal v2.6.3 (90) open reading frame (ORF) calling and DIAMOND v0.8.36 (91)-based homology searches. Loose hits were excluded from the results, and the reference database used was CARD (v2.0.1 release) (6). Results were further separated using RGI’s “perfect” (the predicted gene matches a known curated resistance gene completely at the amino acid level [including SNPs]) and “strict” (above a gene-specific threshold of bitscore-based similarity) credibility levels. Predictions were then grouped and analyzed using CARD’s in-built antibiotic resistance ontology (ARO) and the pandas (v0.22.0) (79) and seaborn (v0.8.1) (80) Python libraries.

The code used to perform this can be found in the notebook and under “analyses/rgi.”

### Comparison of phenotype to genotype.

AST results were compared with the “strict” and “perfect” RGI predictions with and without efflux pump inclusion separately. This was done by using the ARO to identify the class of antibiotics associated with resistance shown by a given detected AMR determinant. These classes were then cross-referenced to the individual antibiotics tested in the AST. If an AMR determinant was detected in a given genome, it was considered to represent a prediction of resistance to the pertinent antibiotics tested. As there were so few isolates with intermediate resistances, all intermediate resistances in the AST were classified as resistant for this and subsequent analyses.

Standard FDA criteria were then used to tally how effective the ‘perfect’ and ‘strict+perfect’ AMR determinants were in predicting the AST as binary presence/absence indicators. These results fell into 3 types (as specific MICs were not being predicted): categorical agreement (CA; the genomic data and AST both predicted susceptibility or resistance); major disagreement (maj; the genomic data predicted resistance but the AST showed susceptibility); and very major disagreement (vmaj; the genomic data predicted susceptible but the AST showed resistance).

Code used to perform this can be found in the notebook and under “analyses/prediction/direct_tallying.”

### Logistic regression.

Antibiotics with either no resistant isolates (azithromycin, ciprofloxacin, gentamicin, nalidixic acid, and trimethoprim-sulfamethoxazole) or an extreme imbalance of resistant/susceptible isolates (chloramphenicol and sulfamethoxazole), defined as the minority class consisting of <5% of isolates, were excluded from the machine learning analyses.

For each of the remaining antibiotics, a simple binary logistic regression model was fitted using the RGI-detected AMR determinants as the input features and “susceptible” and “resistant” as the output labels. Any AMR gene that was found in every isolate was removed from the data matrix. This was performed using scikit-learn v0.20.1 (92). Each model was trained on 80% of the training data (using a stratified test:train split) after resampling was performed using the Synthetic Minority Oversampling Technique (SMOTE) (via imblearn v0.4.3 [93]) to improve label balance. Logistic regression models were tuned using 3-fold cross-validation over the training set, with test-set performance evaluated using precision-recall curves. Performance was then compared across the whole data set using the FDA criteria as described above.

All code used to perform this analysis can be found in the “logistic_regression” folder under “analyses/prediction.”

### Set-covering machine.

In order to assess whether other genomic factors not detected by RGI were likely to contribute to AMR, a k-mer-based set-covering machine approach was applied to the whole genomes for the balanced subset of antibiotics (with exclusion criteria used as described above for logistic regression). This was performed using Kover v2.0.0 (49), and individual rule sets were inferred using 10-fold cross-validation for each of the same antibiotic resistances as were used as described for the logistic regression. The trade-off hyperparameter (p) was selected using cross-validation across a range of possible values from 0.1 to 9999999. A maximum of 10 k-mers were allowed to be included in each rule, and a maximum of 10,000 equivalent rules were output. To assess where the inferred k-mers derived from in the genomes, the inferred equivalent conjunction k-mers were mapped to the genomes using BWA-MEM (94). The resulting individual model SAM files were then analyzed using the PySAM (v0.15.0) library to tally their mapping locations.

All code used to perform this analysis can be found in the “set_covering_machines” folder under “analyses/prediction” in the associated git repository.

### *CMY-2* locus analysis.

The contigs bearing *CMY-2* were annotated using Rapid Annotation using Subsystem Technology (RAST) (95), and the 20-kb regions flanking the *CMY-2* gene were visualized in SEED Viewer version 2.0 (96).

### Relative risk calculation.

Relative risk was calculated from chi-square tests using the SAS software package and the whole data set. The subset of genes selected was determined using the results of the predictive models as well as the *Tet* and *AAC(6’)-Iy* genes that have previously been associated with resistance in the literature.

### Data availability.

All data used in this study are available in GenBank (BioProject identifier PRJNA521409). The full list of accession numbers per genome can be found in Table S1. The code used to perform all analyses is available in the git repository https://github.com/fmaguire/salmonella_ast_prediction/.
