# Supplementary figures and images for: Identification of Primary Antimicrobial Resistance Drivers in Agricultural Nontyphoidal Salmonella enterica Serovars by Using Machine Learning
Source: mSystems. 2019 Aug 6;4(4):e00211-19. doi: 10.1128/mSystems.00211-19 (PMC6687941; doi:10.1128/mSystems.00211-19)

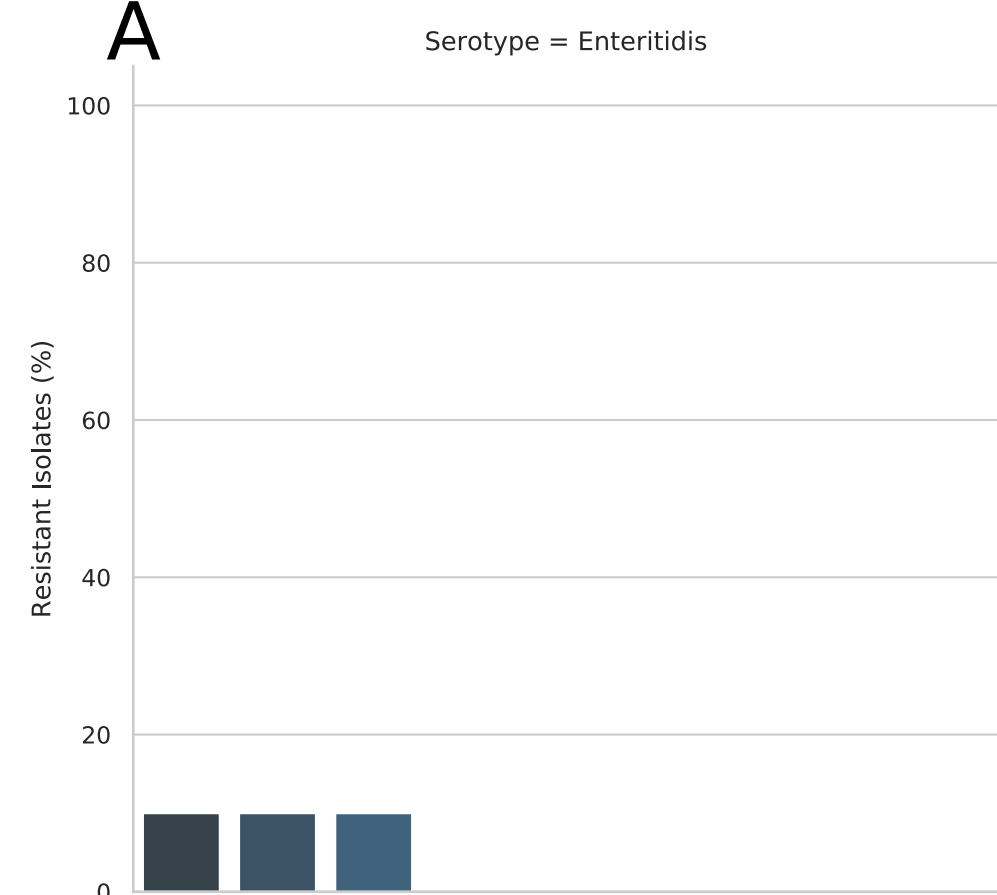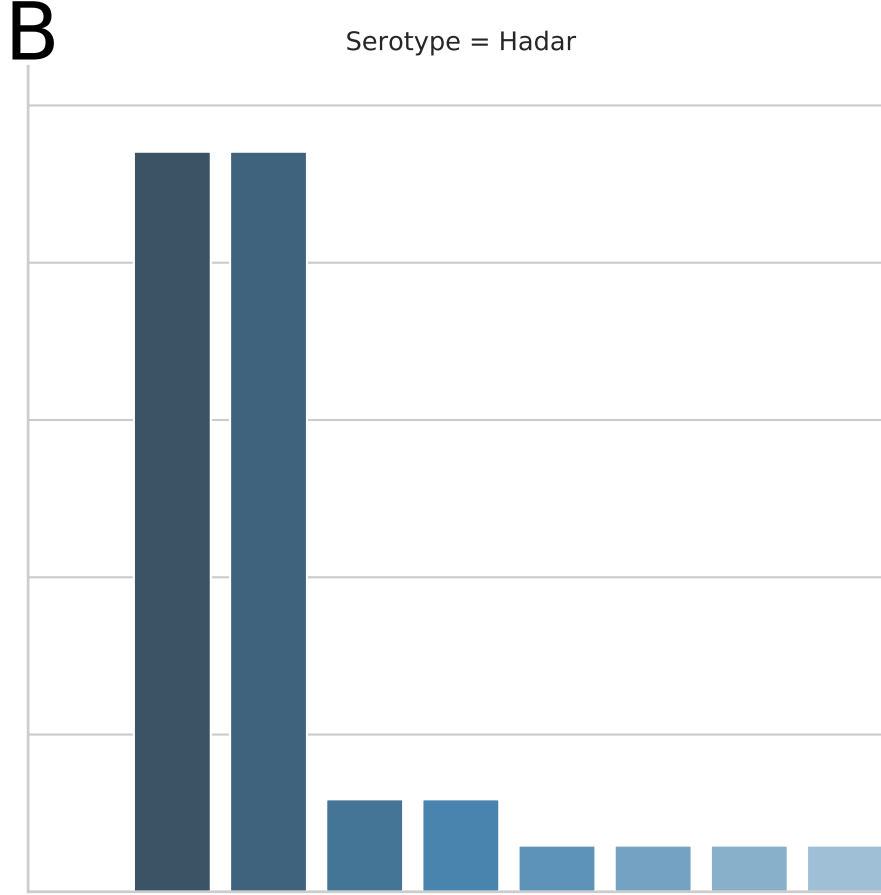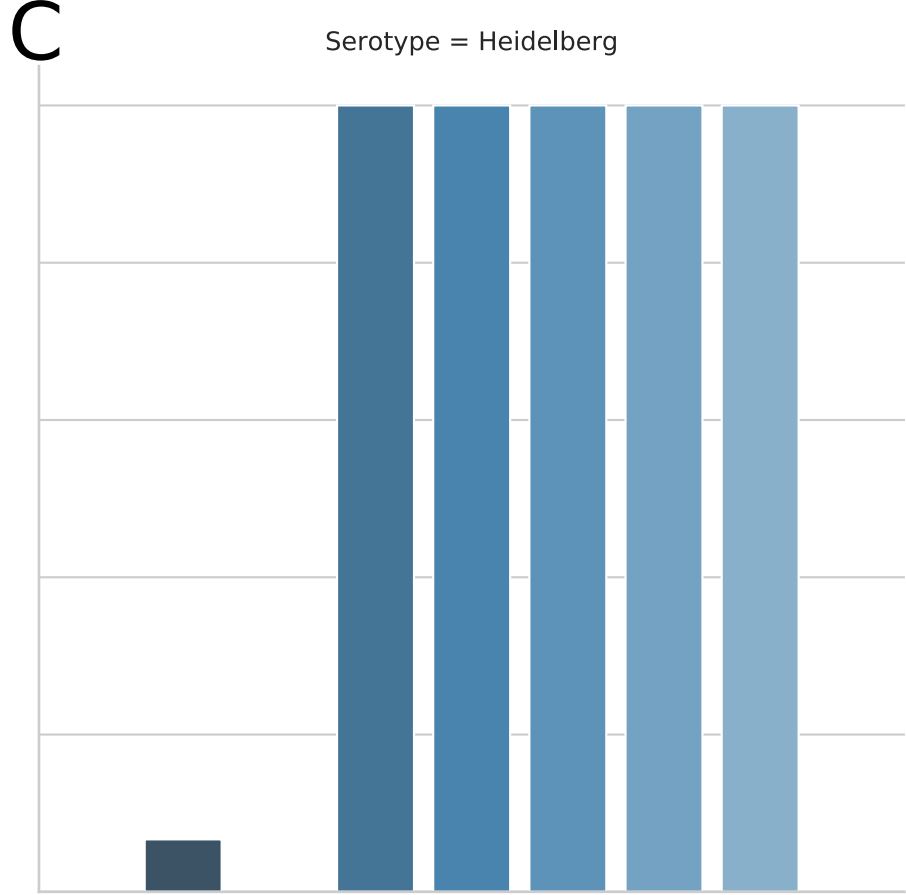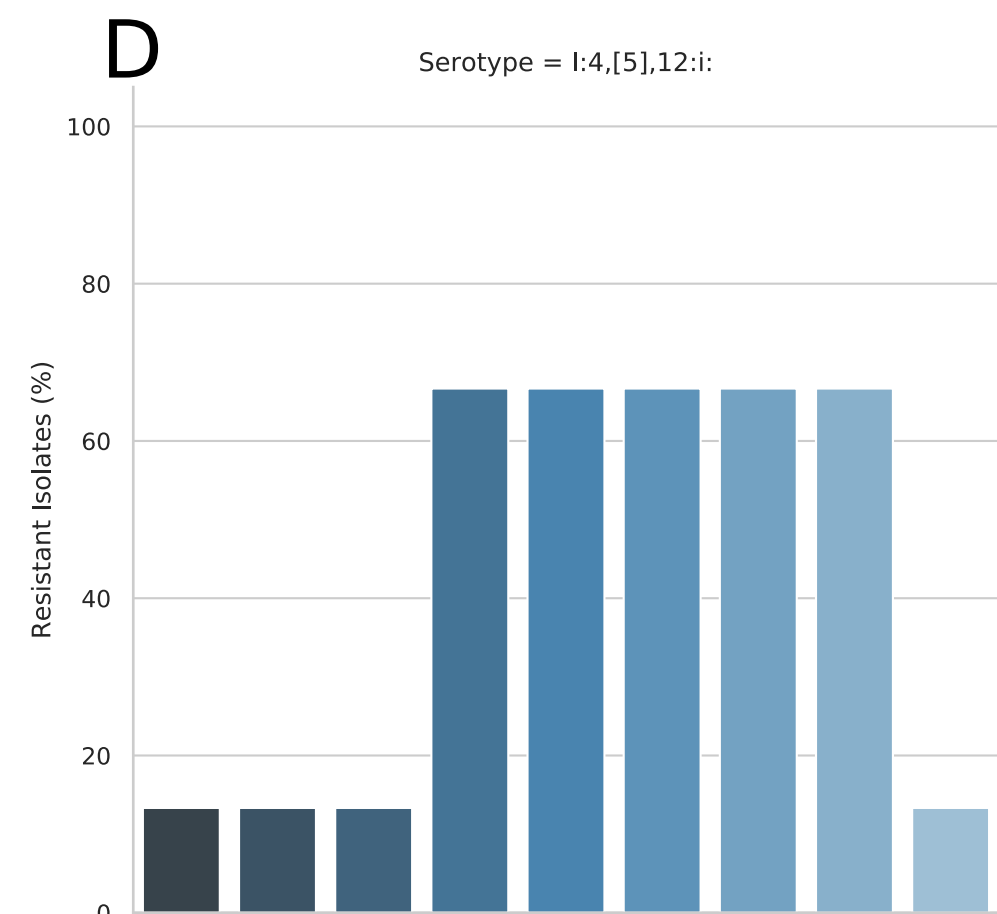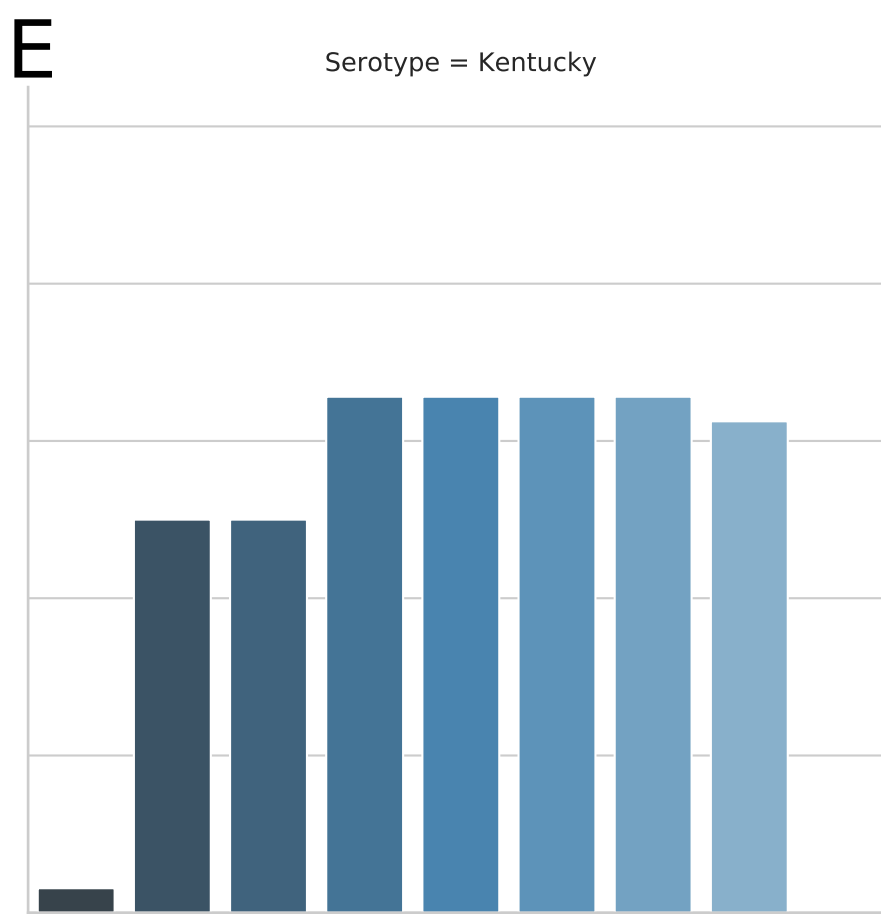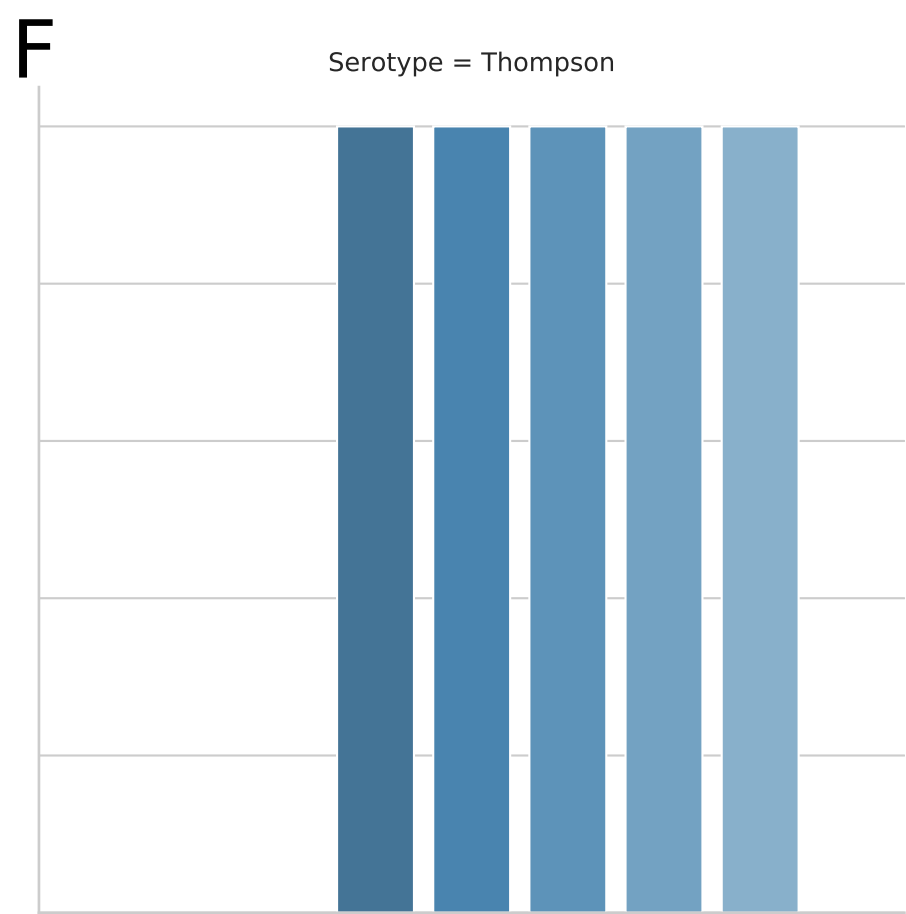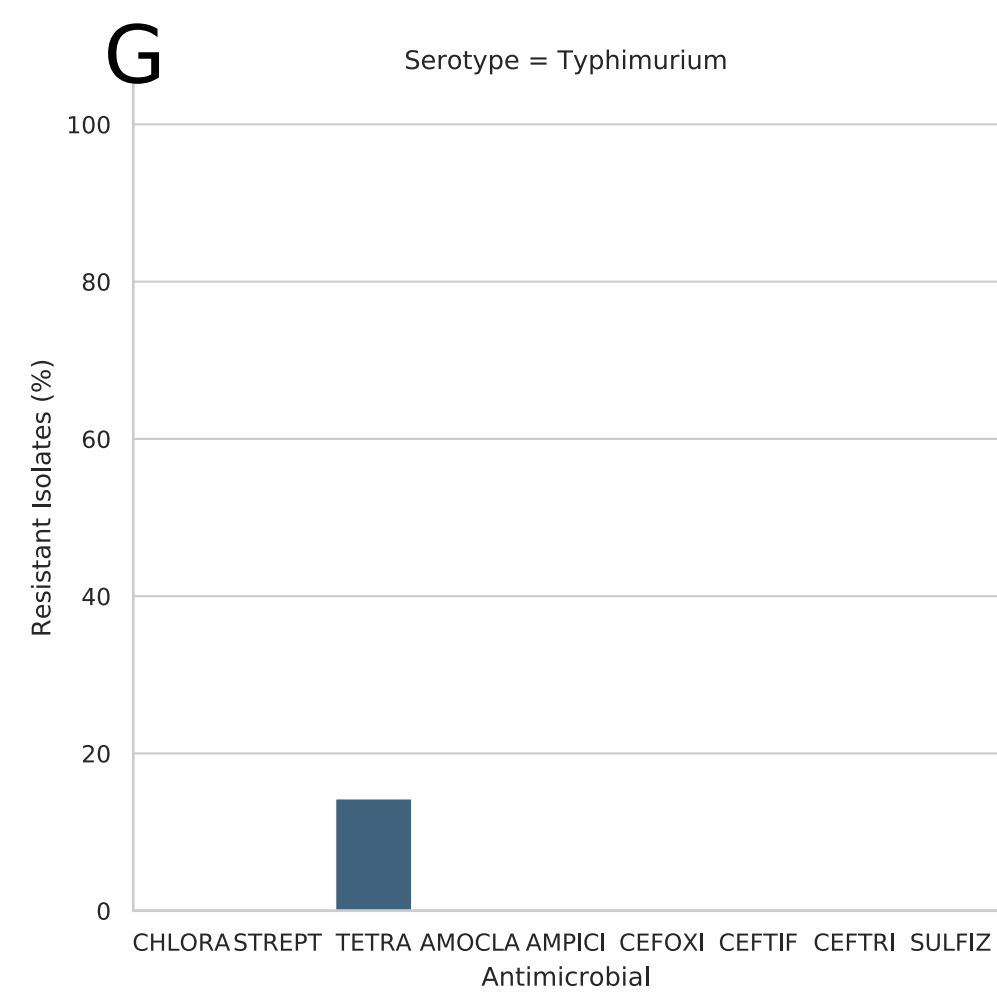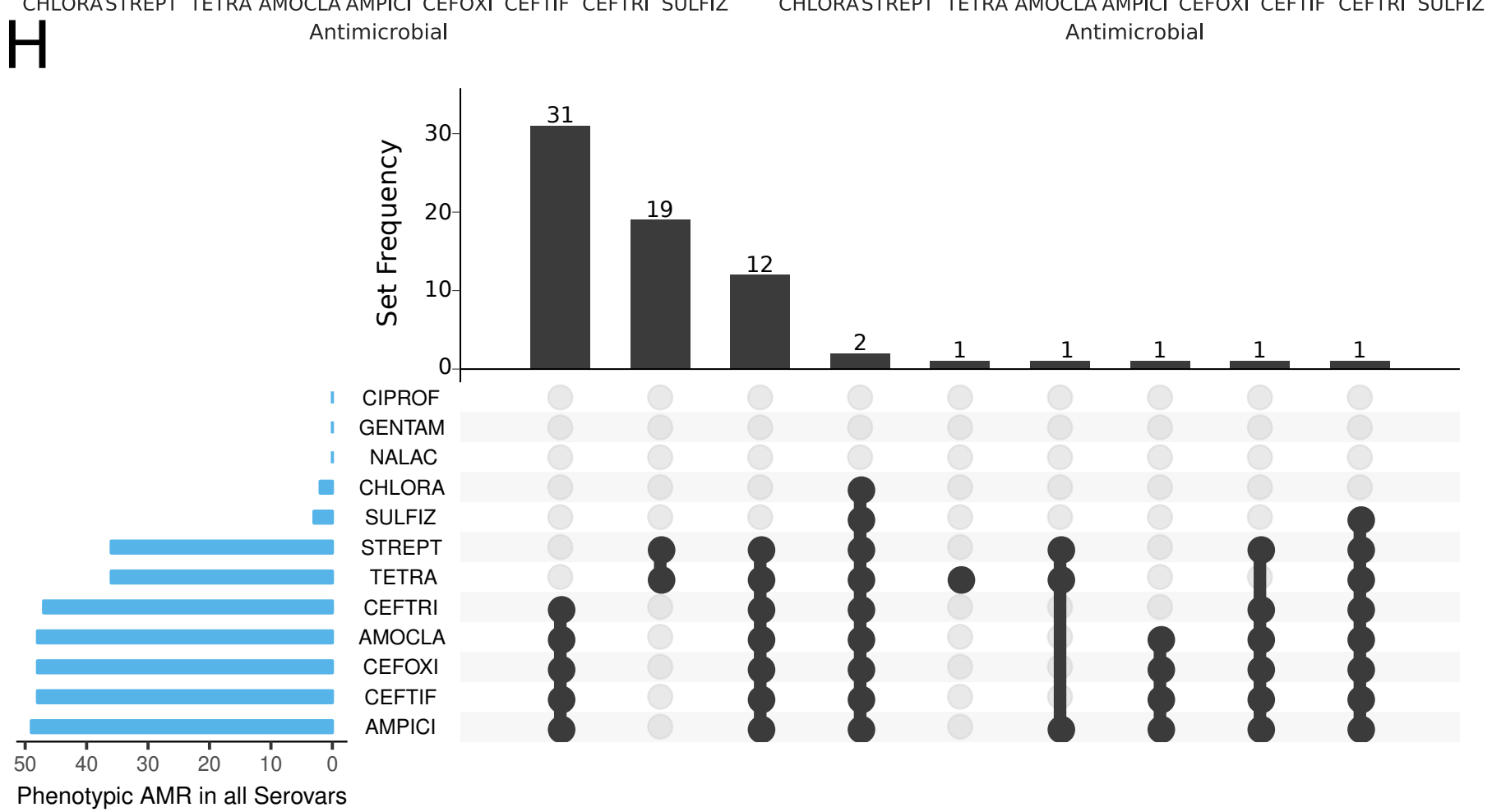

Supplement: FIG S1 [file mSystems.00211-19-sf001.pdf]

Phenotypic AST Status by Genome

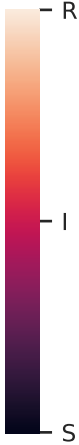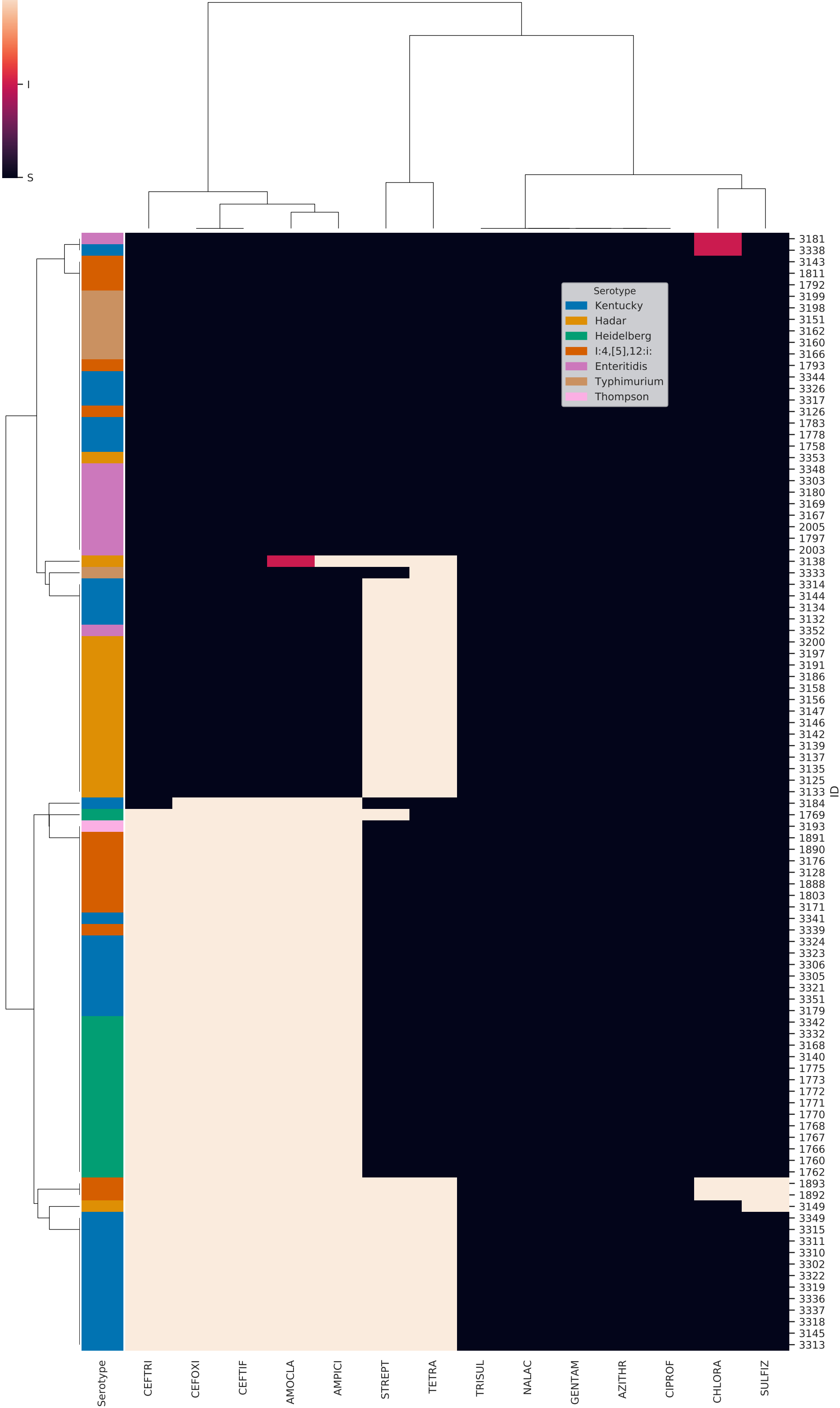

Supplement: FIG S2 [file mSystems.00211-19-sf002.pdf]

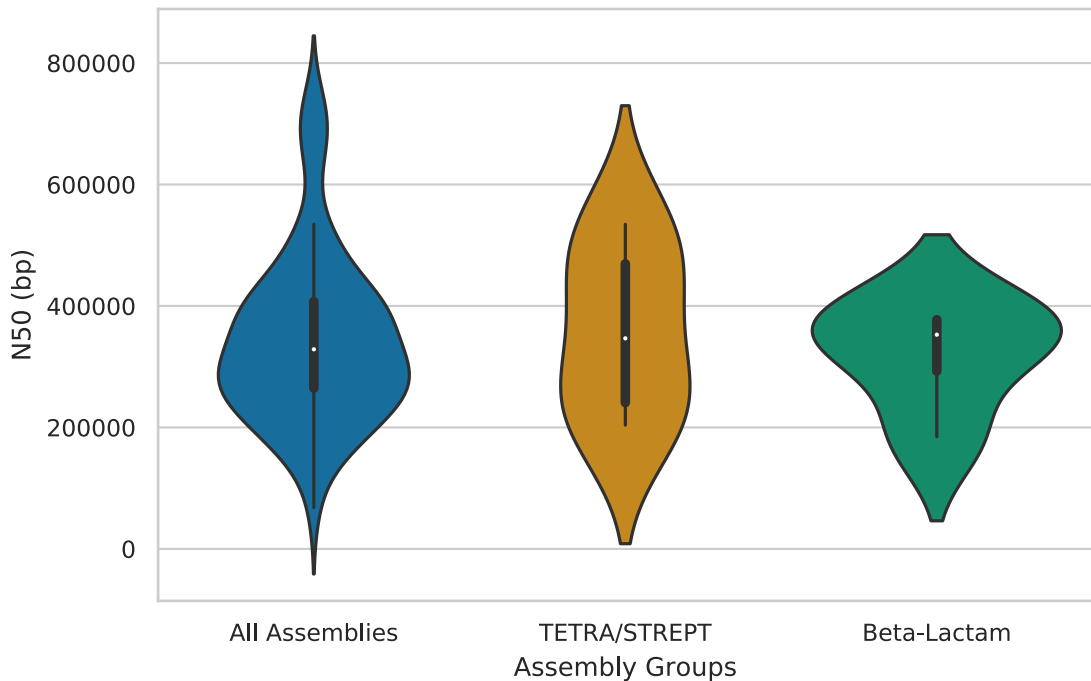

Supplement: FIG S3 [file mSystems.00211-19-sf003.pdf]
