# Supplementary material for: Identification of Primary Antimicrobial Resistance Drivers in Agricultural Nontyphoidal Salmonella enterica Serovars by Using Machine Learning
Source: mSystems. 2019 Aug 6;4(4):e00211-19. doi: 10.1128/mSystems.00211-19 (PMC6687941; doi:10.1128/mSystems.00211-19)
Supplement: FIG S4 [file mSystems.00211-19-sf004.pdf]

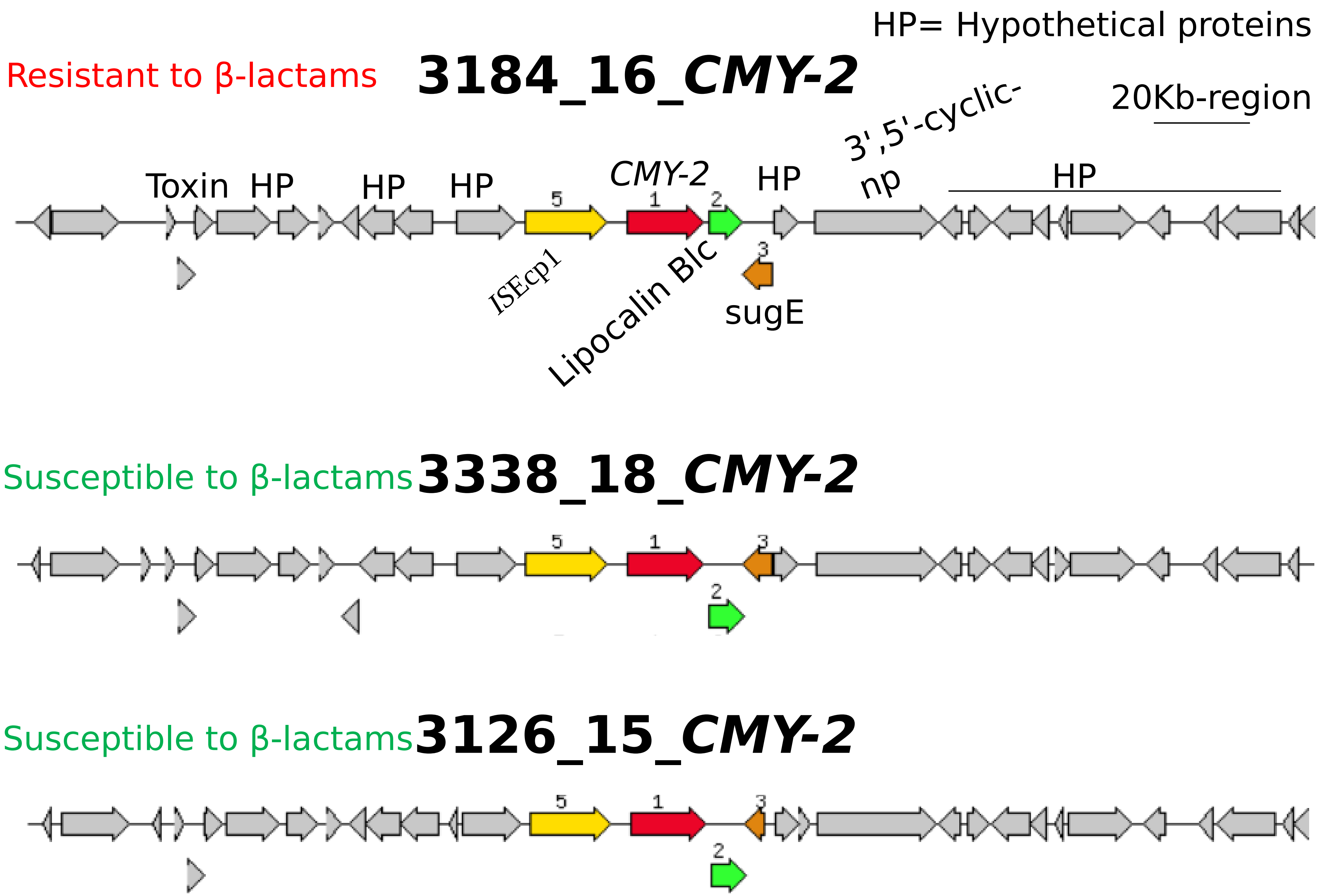
HP= Hypothetical proteins
Resistant to β-lactams
3184_16_CMY-2
20Kb-region
Toxin
HP
HP
HP
CMY-2
HP
3',5'-cyclic-
np
HP
5
1
2
3
ISEcp1
Lipocalin Blc
sugE
Susceptible to β-lactams
3338_18_CMY-2
5
1
3
2
Susceptible to β-lactams
3126_15_CMY-2
5
1
3
2
