# Supplementary material for: Identification of Primary Antimicrobial Resistance Drivers in Agricultural Nontyphoidal Salmonella enterica Serovars by Using Machine Learning
Source: mSystems. 2019 Aug 6;4(4):e00211-19. doi: 10.1128/mSystems.00211-19 (PMC6687941; doi:10.1128/mSystems.00211-19)
Supplement: FIG S5 [file mSystems.00211-19-sf005.pdf]

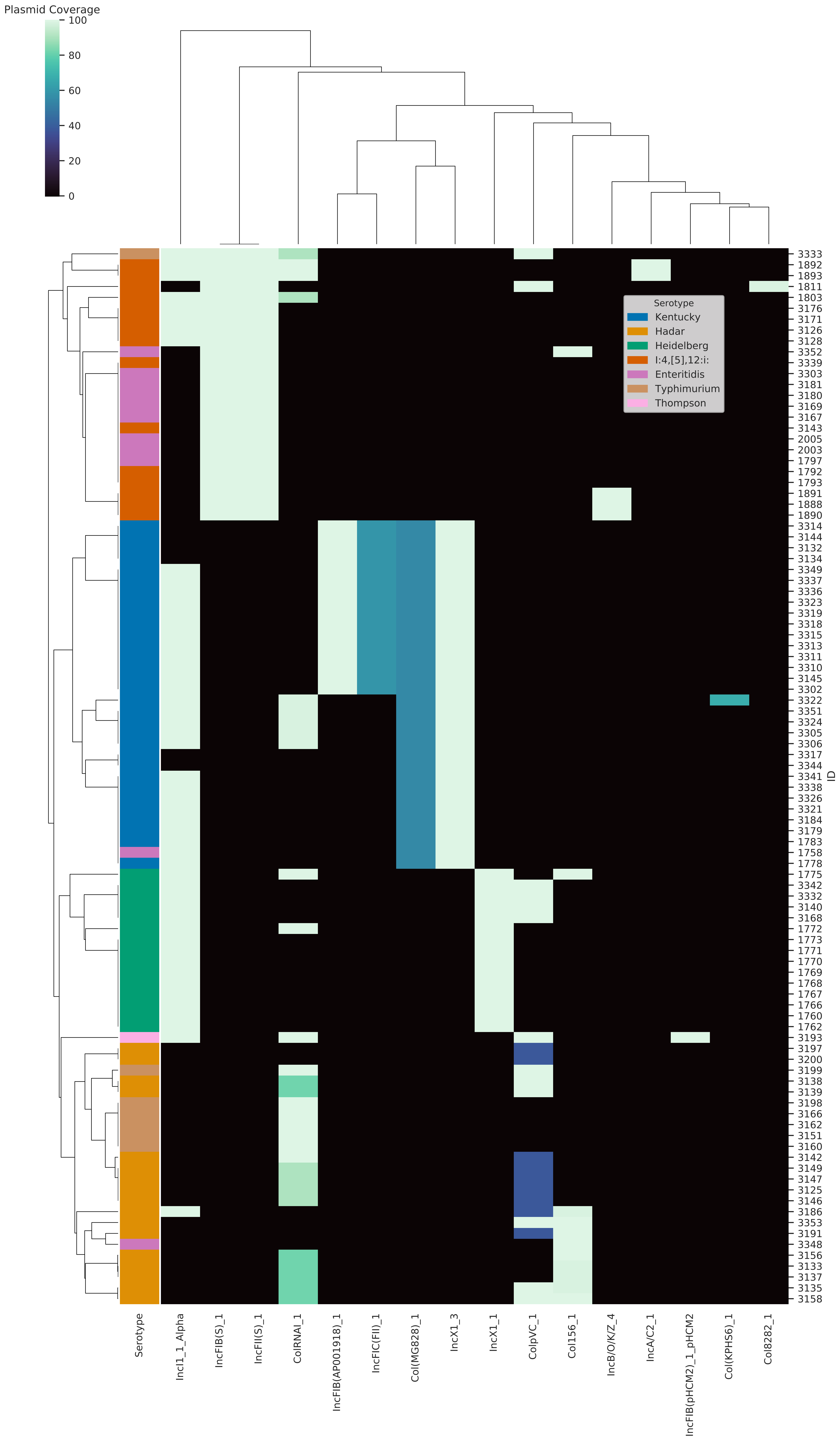

Plasmid Coverage
100
80
60
40
20
0
Serotype
Kentucky
Hadar
Heidelberg
I:4,[5],12:i:
Enteritidis
Typhimurium
Thompson
ID
3333
1892
1893
1811
1803
3176
3171
3126
3128
3352
3339
3303
3181
3180
3169
3167
3143
2005
2003
1797
1792
1793
1891
1888
1890
3314
3144
3132
3134
3349
3337
3336
3323
3319
3318
3315
3313
3311
3310
3145
3302
3322
3351
3324
3305
3306
3317
3344
3341
3338
3326
3321
3184
3179
1783
1758
1778
1775
3342
3332
3140
3168
1772
1773
1771
1770
1769
1768
1767
1766
1760
1762
3193
3197
3200
3199
3138
3139
3198
3166
3162
3151
3160
3142
3149
3147
3125
3146
3186
3353
3191
3348
3156
3133
3137
3135
3158
Serotype
IncI1_1_Alpha
IncFIB(S)_1
IncFII(S)_1
ColRNAI_1
IncFIB(AP001918)_1
IncFIC(FII)_1
Col(MG828)_1
IncX1_3
IncX1_1
ColpVC_1
Col156_1
IncB/O/K/Z_4
IncA/C2_1
IncFIB(pHCM2)_1_pHCM2
Col(KPHS6)_1
Col8282_1
